# Non-surgical management for air injection injury to the hand, a case report

**DOI:** 10.1080/23320885.2022.2114480

**Published:** 2022-08-28

**Authors:** William Pipkin, Alex Frangenberg, Michael Wade, Weston Peine, William F. Pientka

**Affiliations:** a Department of Orthopaedic Surgery, JPS Health Network, Fort Worth, TX, USA; b School of Medicine, University of North Texas Health Science Center, Fort Worth, TX, USA

**Keywords:** Hand injection injuries, air injection injury

## Abstract

We present a case a high pressure air injection injury to the index finger with air extension proximal to the elbow. This patient was treated non-surgically with close observation. At 3 year follow-up, no lasting deficits or complications were noted and radiographs revealed complete resolution of the air tissue dissection.

## Introduction

High pressure injection injuries are rare and typically occur in industrial settings with water, or caustic organic agents such as paint, grease, diesel and jet fuel. These injuries vary in degree of severity depending on the pressure and substance that is injected. These types of injuries typically present with minor soft tissue trauma at the injection site, most often with mild pain and swelling in the digits or palmar aspect of the hand. Under such high pressure, the offending agent often extends proximally transecting fascial planes [1]. Due to pain being delayed or absent, it is common for patients to present on a delayed basis after these injuries, thus enhancing the probability of having more severe complications from the injection. These complications include decreased range of motion, loss of strength, and decreased manual dexterity in the affected limb with high rates of infection, tissue necrosis, and subsequent amputation [2].

Current treatment consists of tetanus prophylaxis, broad spectrum antibiotics, and urgent hand surgeon consultation for operative debridement. Improved outcomes are seen when treatment is initiated within six hours of injection injury [3].

## Case report

**Figure 1.**
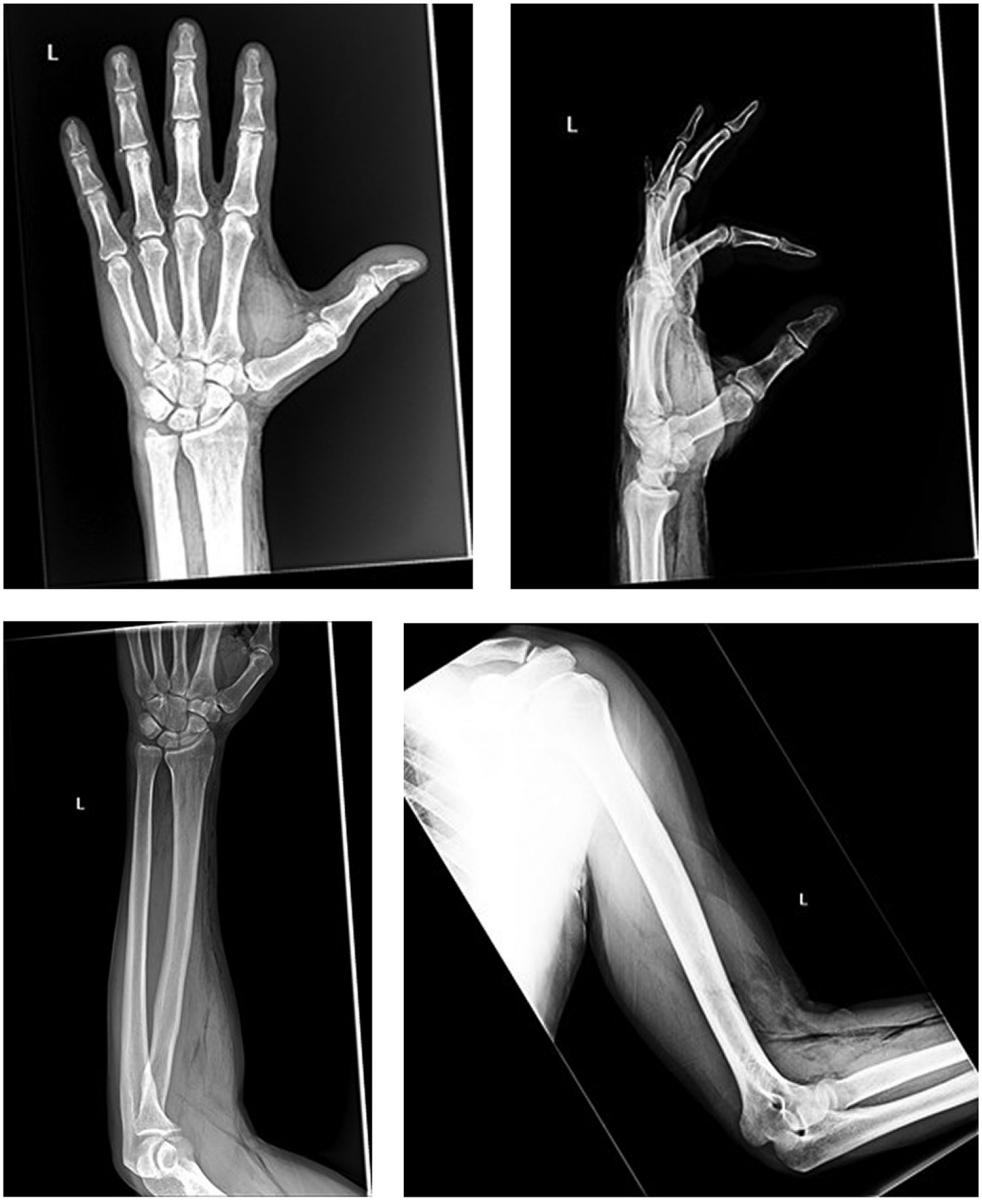
X-rays of left hand, wrist, forearm and humerus showing extensive subcutaneous emphysema throughout the hand, wrist, forearm, and arm.

A 54-year-old mechanic presented to the emergency department 5 days after suffering a high pressure air injection injury to the radial base of the index finger through a pre-existing finger laceration. He presented for increased swelling and pain along the hand and forearm. The pressure of the air line and time of exposure to the high pressure were unknown. The patient was hemodynamically stable on arrival. Physical examination demonstrated a 1 cm laceration on the radial aspect of the index finger base with subtle erythema. There was significant stiffness to the index finger. There was also mild swelling on the dorsum of the hand and forearm. The digits, hand, and forearm were neurovascularly intact and there was no evidence of flexor tenosynovitis, compartment syndrome, or carpal tunnel syndrome. There was no obvious subcutaneous emphysema on exam. White blood cell count, erythrocyte sedimentation rate, and C-reactive protein were within normal limits. Radiographs of the left upper extremity (Figure 1) revealed extensive subcutaneous emphysema throughout the hand, wrist, forearm and arm dissecting along fascial planes.

**Figure 2.**
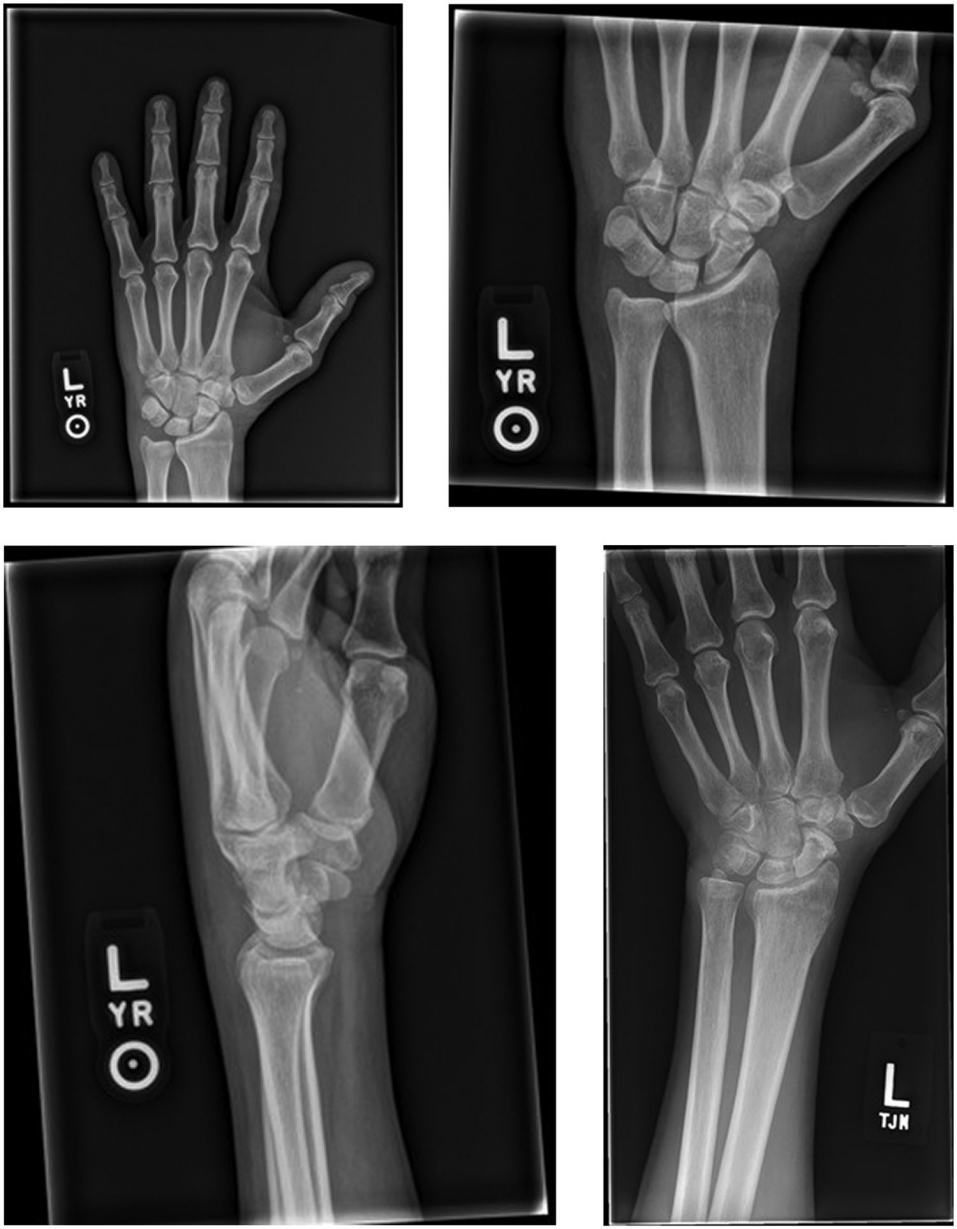
X-rays of the left hand and wrist at 2.5 year follow-up showing resolution of the subcutaneous air with no obvious sequelae. A new unrelated scaphoid fracture is noted.

The patient was discharged on a 10 day course of Augmentin with strict emergency department precautions and scheduled for follow up in the Orthopaedic clinic. The patient recovered uneventfully without evidence of infection or complication. He subsequently presented back to the emergency department approximately 2 and a half years later after falling off of a ladder and suffering a left scaphoid fracture. X-rays of his left hand and wrist (Figure 2) demonstrated resolution of all subcutaneous air.

## Discussion

Outcomes for high pressure injection injuries vary immensely depending on the substance injected. Typically, industrial solvents and chemical injections induce more intense engagement of inflammatory and necrotic properties of the tissues, subsequently necessitating surgical intervention [3]. For injections resulting in less damage to the local anatomy, a conservative intervention involving antibiotics, steroid, tetanus toxoid, elevation, and close observation may be utilized with good outcomes [4]. Evaluation for compartment syndrome or acute carpal tunnel syndrome as a possible emergent problems should be considered [5]. In the case presented here, the patient presented 5 days after the injection injury without symptoms consistent with compartment syndrome or acute carpal tunnel syndrome, and thus there was no place for emergent or even urgent debridement. Despite the delayed presentation and non-surgical treatment, the patient recovered completely without complication given the non-caustic nature of the injection.

Pneumomediastinum may be found with high pressure air injection which may be managed conservatively once esophageal or tracheo-bronchial perforation has been ruled out. Injection of liquids (paint, grease, jet fuel, or other caustic substances) has been shown to have substantially high rates of infection and require emergent surgical debridement. Mirzyan et al. reported positive cultures of gram-negative organisms in 15 of 32 patients with increased need for amputation up to 50% in the case of oil-based paint injection [6]. In such instances, there is a correlation between shorter time from injury to surgery and improved prognosis [7]. Various literature has demonstrated amputation is not necessary with air injection injuries [8,9]. Also, it is indicated that although high-pressure injections are dangerous and destructive, air injections are less destructive than injections of more caustic substances and interventions such as aggressive fasciotomy and debridement should not be rushed [9].

Injection injuries highlight the importance of physical exam and thorough patient history. This infrequently encountered injury frequently presents with mild symptoms that can be misdiagnosed, resulting in severe, debilitating consequences for the patient. Long-term sequelae of injection injuries to the hand reported in a group of patients include significant stiffness, prolonged recovery of greater than 6 months, and inability to return to their previous level of employment [2]. As demonstrated in this case, however, air injection injury can resolve with non-operative management if surgical emergencies are appropriately excluded.
